# Stress reactivity of the autonomic nervous system in youth with and without major depressive disorder

**DOI:** 10.1007/s00787-026-02998-5

**Published:** 2026-03-25

**Authors:** Nikola Fann, Anne Martinelli, Helena Oldenhof, Christine M. Freitag, Anka Bernhard

**Affiliations:** 1 Department of Child and Adolescent Psychiatry, Psychosomatics and Psychotherapy, University Hospital Frankfurt am Main, Goethe University, Frankfurt am Main, Germany; 2 Fresenius University of Applied Sciences Frankfurt am Main, Frankfurt am Main, Germany; 3 Amsterdam UMC, Vrije Universiteit, Amsterdam, The Netherlands; 4 Department of Child and Adolescent Psychiatry and Psychotherapy, Faculty of Medicine, German Center for Child and Adolescent Health (DZKJ), Technische Universität Dresden, partner site Leipzig/Dresden, Fetscherstraße 74, Dresden, D-01307 Germany

**Keywords:** Major depressive disorder, Autonomic nervous system, Respiratory heart rate variability, Pre-ejection period, Sex differences, Adolescents

## Abstract

Altered Autonomic Nervous System (ANS) function has been described in youth with Major Depressive Disorder (MDD), but studies are scarce and rarely consider sex, nor both parasympathetic and sympathetic ANS measures. Stress regulation plays a key role in the emergence and maintenance of MDD. Here, we aimed to investigate ANS stress reactivity within a clinical sample of female and male youth with MDD compared to healthy controls (HCs). Parasympathetic and sympathetic ANS stress reactivity (Heart Rate, HR; Respiratory Heart Rate Variability, RespHRV; Pre-ejection Period, PEP) was studied in response to a standardized psychosocial stress task (Trier Social Stress Test) in *n* = 100 youth with MDD (60% female; 12–18 years), and *n* = 70 HCs, while controlling for relevant confounders (e.g., age, puberty). Besides higher levels of self-reported psychological stress, youth with MDD showed lower HR increase, lower parasympathetic (less increase of RespHRV), and lower sympathetic (less reduction of PEP) ANS stress response compared to HCs. No effects of sex were found. Findings indicate blunted parasympathetic and sympathetic ANS stress responses contrasting stronger psychological stress experience in both female and male youth with MDD compared to HCs. This altered psychophysiological stress response may provide valuable insights for the development and optimization of future diagnostic and therapeutic approaches for youth with MDD. Further studies are needed to investigate the underlying biological backgrounds and longitudinal trajectories to understand the influence on stress regulation on onset and remission of MDD.

**Supplementary Information:**

The online version contains supplementary material available at 10.1007/s00787-026-02998-5.

## Introduction

Over the past years, the prevalence of major depressive disorder (MDD) in youth has increased [1], accompanied by strong impairments in daily functioning and a high risk of severe comorbid mental disorders [2] or mortality [3]. While less frequent in childhood, the prevalence of MDD rises sharply throughout adolescence to a worldwide 1-year prevalence in late adolescents above 4% [1]. Given the increase of MDD prevalence in youth, it is crucial to elucidate underlying contributing factors and associated neurobiological alterations. As one of the major systems involved in stress regulation, altered autonomic nervous system (ANS) activity has previously been interpreted as a maladaptive mechanism connected to MDD [4]. As the ANS continues to develop during youth [5], it may be particularly vulnerable to dysregulation, indicating clinical relevance for ANS research in youth with MDD. Treatment strategies based on ANS alterations in youth with MDD and their modification derive from research linking ANS alterations and MDD [6], yet literature on associations between specific ANS components and MDD in youth are inconsistent.

The ANS consists of two anatomically separate, but functionally linked pathways: the (1) parasympathetic nervous system (PNS), dominating during rest, and (2) sympathetic nervous system (SNS), activated in response to external or internal threats [7]. Various cardiovascular measurements serve as markers of ANS activity. In particular, heart rate (HR) is a most easily measurable marker influenced by both PNS and SNS functioning thereby reflecting the combined autonomic influence exerted on cardiac activity. As PNS and SNS indices contribute relatively equally to HR responses to stress [8] specificity regarding the relative influence of each pathway should be considered. In response to a standardized psychosocial stress task, HR was found to increase in healthy youth and adults [9], while remaining attenuated in adults with MDD [10]. Remarkably, studies on HR stress reactivity in youth with MDD are lacking.

A widely used indicator for PNS activity is heart rate variability, often assessed by non-invasive measurement of respiratory sinus arrythmia (RSA) [11–13]. Recent reporting guidelines encourage referring to RSA as RespHRV [14]. We therefore use RespHRV as equivalent to RSA. Higher RespHRV values indicate greater PNS activity. Studies on PNS reactivity in female and male youth with MDD are limited, characterized by conflicting results [15–17]. Some studies reported greater PNS than SNS activation in female and male youth with a history of or current MDD, whereas healthy controls (HCs) showed the opposite response pattern to an unsolvable puzzle or stress inducing Go/NoGo task [16, 18]. In another study, no differences in PNS stress response in female and male youth with and without MDD were found [15], while a fourth study reported a stress-related RespHRV decrease in HCs but not youth with a history of MDD [19]. Findings in adults with MDD are more homogeneous across several studies, reporting blunted RespHRV responses to psychosocial stress [10].

For the SNS, the Pre-ejection Period (PEP) serves as a valid non-invasive marker [11, 12] with lower values indicating greater SNS activity. As the SNS serves as the body’s major “fight or flight” system [7], its consideration is relevant when investigating stress responses. A recent meta-analysis using community and clinical samples found a PEP decrease (i.e., increase in SNS activation) in response to a psychosocial stress task, indicating SNS activation as a functional physiological stress reaction [9]. However, studies in youth with MDD are heterogeneous, reporting lower [16], higher [18] or no differences in PEP stress reactivity compared to HCs during psychological or physiological stress-inducing tasks [19]. In adults, blunted sympathetic stress response to a speech stressor task was found for MDD [20].

Finally, given the higher incidence and prevalence of MDD in girls compared to boys [1, 2], sex differences also need to be considered. Healthy girls showed higher HR increase [21, 22] and altered PNS parameters [23] in response to stress than boys. In MDD, recent findings suggest boys experience a more severely altered ANS reactivity (i.e., cardiac autonomic balance) than girls [18].

Taken together, previous work on the ANS stress response in youth with MDD is scarce, and characterized by inconsistent findings, clinically heterogenous study populations (i.e., youth with MDD, MDD symptoms, history of MDD), and diverging methods (e.g., cold pressor test, sad film clips, Go/NoGo or unsolvable puzzle tasks). Also, the influence of sex or potentially confounding factors has been neglected. Furthermore, while previous research has largely focused on PNS stress reactivity, SNS and HR stress response have rarely been investigated, despite their functional linkage [7]. The current study therefore aimed to investigate both PNS and SNS (i.e., HR, RespHRV, PEP) response to a standardized psychosocial stress task (Trier Social Stress Test [24]) in a clinical sample of female and male youth with MDD compared to HCs. Based on consistent findings of blunted HR, PNS, and SNS reactivity in adults with MDD [10, 20], we hypothesized an overall reduced ANS response to psychosocial stress in youth with MDD compared to HCs. Furthermore, in line with recent findings suggesting a more severely altered ANS response in boys compared to girls with MDD [18], we hypothesized a more blunted ANS response in boys compared to girls with MDD.

## Methods

### Participants

This study included *n* = 100 youths with MDD (60% female) and *n* = 70 HCs (61% female) aged 12 to 18 years (Mean [SD]: MDD 15.35 [1.47], HCs 15.00 [1.66]). Most participants were born in Germany (94%). Data were collected between 2014 and 2018 at the Goethe University Hospital Frankfurt am Main, Germany. Participants with MDD were recruited from the clinic’s day-care and inpatient wards within a local study focusing on adolescent depression. HCs from the community were included from the Frankfurt part of the “FemNAT-CD” study [25] according to available stress response data and suitability regarding age and sex for comparison with the MDD sample. MDD participants fulfilled DSM-IV-TR criteria for a current major depressive episode. HCs did not have any current mental health disorder but were not excluded for any lifetime mental disorder. Exclusion criteria for both groups included IQ < 70, pregnancy, pre-pubertal status, last menstruation > 6 months, and a history of neurological disorder, known genetic disorders, traumatic brain injury, or DSM-IV-TR diagnosis of schizophrenia, autism spectrum disorder, disruptive behavior disorder, and current mania or bipolar disorder (or history of in HCs). Prior to participation, all youth and their legal guardians gave written informed consent after detailed study explanation.

## Procedures

As in earlier “FemNAT-CD” publications [26], current and past mental disorders were assessed by the semi-structured interview Kiddie-Schedule for Affective Disorders and Schizophrenia -Present and Lifetime Version (K-SADS-PL), IQ with the Wechsler Intelligence Scales, pubertal status with the Pubertal Development Scale, and parental educational status using the International Standard Classification of Education criteria. Weight and height were measured to calculate BMI, and smoking, exercise, and medication were assessed via self-report. For detailed description of all procedures, see Online Resource 1.

## Trier social stress test and psychological stress response

**Fig. 1:**
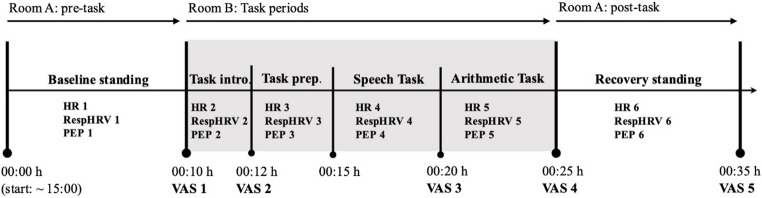
Time schedule of the applied procedure. *Note*: After a standing relaxation period in comfortable Room A, participants entered a sparsely equipped Room B where they were introduced to the task and left alone for task preparation. The speech task started with entering of two for the participant unknown jurors in Room B. After the mental arithmetic task, participants entered again Room A where they were debriefed (for details see Online Resource 1). HR=heart rate, PEP = pre-ejection period, RespHRV=respiratory heart rate variability, Task intro.=task introduction, Task prep.=task preparation, VAS=visual analogue scale

The Trier Social Stress Test (TSST) was applied as a widely used, reliable, and valid method for inducing acute psychosocial stress [9, 27, 28] covering a public speech and mental arithmetic task in front of an unknown panel (see Fig. 1, Online Resource 1). Psychological stress was measured five times during the stress procedure applying a Visual Analogue Scale (”Do you feel stressed?”, range 0=“no, not at all” to 10=”yes, very much”) [29].

## Autonomic nervous system measurement

During the TSST, participants were connected to the Vrije Universiteit Ambulatory Monitoring System (VU-AMS) device recording electrocardiography (ECG) and impedance cardiography (ICG) data [11, 30, 31]. The Vrije Universiteit Data Analysis and Management software package (VU-DAMS 4.3) was used for generating the raw data output of the recorded ECG and ICG [30, 31]. HR was calculated through the R-peak time series of the ECG (beats per minute, bpm). RespHRV scores were derived from the recorded ECG. For respiration data, the shortest interbeat interval during inspiration was subtracted from the longest interbeat interval during expiration [11]. PEP is defined as the time between the beginning of ventricular depolarization marked by the Q-wave onset in the ECG and the opening of the aortic valve marked by the B-point in the ICG [32]. Higher relative HR (bpm) reflects a domination of sympathetic activity over parasympathetic activity and vice versa. Higher RespHRV values (msec) reflect higher parasympathetic activity whereas lower PEP values (msec) reflect higher sympathetic activity (Online Resource 1).

## Statistical analyses

Statistical analyses were performed using SPSS v29 (IBM Corp., Armonk, NY) with two-tailed tests and significance level of *p*<.05. Demographic and clinical characteristics were compared using univariate analysis of variance or Chi-Squared-tests. To investigate Spearman correlations of perceived psychological stress and ANS measures, corresponding areas under the curve with respect to increase (AUC_I_) were calculated [33].

Four separate repeated-measures analyses of covariance (rmANCOVA) were performed to investigate ANS stress responses of the dependent variables psychological stress, HR, RespHRV, and PEP with time as the within-subject factor along with group (MDD vs. HCs) and sex (female vs. male) as between-subject factors. RespHRV measures (right-skewed) were ln-transformed and PEP measures (left-skewed) reversely ln-transformed to normalize their distribution. Covariates for ANS analyses included age, pubertal status, body mass index (BMI), regular exercise (hours per week), and smoking (yes/no) given their previously reported influence on the ANS [22, 34, 35]. The rmANCOVA on psychological stress was controlled for age and pubertal status. Age, pubertal status, BMI, and exercise measures were mean centered [36]. To control for respiration rate on RespHRV measures [37], RespHRV analyses were re-run with an adjusted RespHRV variable covering the standardized residuals from the linear regression of the RespHRV and respiration rate for each time period. Where necessary, Greenhouse-Geisser corrections were applied while the uncorrected degrees of freedom are presented for clarity in all tables. Significant differences in ANS stress responsivity were followed by post-hoc Bonferroni-corrected pair-wise comparisons. Effect sizes are reported using partial eta squared (η²_p_≥.01 small, η²_p_≥.06 medium, and η²_p_≥.14 large effects) [38]. With a power of 80% and a two-tailed significance level of *p*<.05, our a-priori determined sample size allowed us to detect small effects (*f*<0.20, respectively Cohen’s *d*<0.40) for between and within factors as well as within-between interactions of group, sex, and time for five and six measurements based on conservative rmANOVA estimation (G*Power 3.1.9.7).

Sensitivity analyses investigated the possible influence of comorbid mental disorders and medication by additionally including one of the following covariates in each rmANCOVA: ADHD, anxiety disorders, post-traumatic stress disorder (PTSD), eating disorders, antipsychotic medication, selective serotonin reuptake inhibitor (SSRI) medication, and other medication (e.g., asthma medication, pain killer). Because of the high prevalence of comorbid lifetime anxiety disorder and/or PTSD (ANX/PTSD) in the MDD group, we furthermore performed group separated rmANCOVAs for the psychological and ANS stress response for participants with MDD with (+) and without (-) lifetime anxiety disorder and/or PTSD compared to HCs without (-) lifetime anxiety disorder and/or PTSD (1. MDD + ANX/PTSD vs. HC–ANX/PTSD and 2. MDD–ANX/PTSD vs. HC–ANX/PTSD). To follow up possible effects of SSRI/antidepressant intake, additional rmANCOVAs for the psychological and ANS stress response were performed comparing participants with MDD with and without current SSRI/antidepressant medication to HCs. Furthermore, we conducted an independent sample t-test determining possible group differences (MDD+SSRI vs. MDD-SSRI) on MDD severity. Additionally, rmANCOVAs for ANS measures (HF, RespHRV, and PEP) were conducted with psychological stress as an additional covariate.

## Results

### Sample characteristics

Groups did not differ in sex, age, pubertal status, estimated IQ, exercising, and parental educational status (see Table 1). Youth with MDD showed higher BMIs and rates of current smoking, medication use (antipsychotics, SSRIs/antidepressants, other), depressive symptoms, and comorbid mental disorders (ADHD, anxiety disorders, PTSD, eating disorders). Most female participants (MDD 98.3%, HCs 90.7%) had already experienced menarche, and no significant group difference was observed in the number of days since their last menstruation (Mean [SD]: MDD 18.38 [10.96], HCs 18.38 [8.92], *p* = 1.00; 87.4% complete information). Few female participants took hormonal contraception (MDD 17.5%, HCs 16.2%) with no significant difference between groups (*p*=.88; 91.3% complete information). Most MDD participants were inward patients (57% inward treatment, 16% day clinic treatment, 16% no treatment, 5% outpatient treatment, 3% emergency treatment, 3% unknown) with no significant difference in MDD severity between MDD treatment groups (mean [SD]: Total = 7.18 [9.18], *p*=.47).

During the pre-task standing period (baseline), youth with MDD showed higher psychological stress (mean [SD]: MDD = 3.11 [3.05], HCs = 0.88 [1.49], *p*<.001) and lower RespHRV (msec; mean [SD]: MDD = 38.55 [19.79], HCs = 47.53 [22.11], *p*=.01) than HCs. No group differences were found for baseline HR (bpm; mean [SD]: MDD = 93.61 [11.73], HCs = 93.06 [12.66], *p*=.77) nor baseline PEP (msec; mean [SD]: MDD = 110.67 [16.07], HCs = 114.02 [10.42], *p*=.13). Girls showed higher levels of baseline HR than boys (bpm; mean [SD]: females = 95.18 [12.06], males = 90.48 [11.76], *p*<.01). No other sex or group-by-sex interaction effects for baseline psychophysiological measures were found.

**Table 1:** Sociodemographic and descriptive characteristics of the sample

	Female (n=103)		Male (n=67)		Group	Sex	Group x sex
	MDD (*n *= 60)	HCs (*n* = 43)	MDD (*n* = 40)	HCs (*n* = 27)	*p*	*p*	*p*
Age	15.22 (1.52)	15.00 (1.77)	15.55 (1.38)	15.00 (1.50)	.12	.50	.50
Pubertal status					.07	<.001	
Mid-pubertal	1 (1.67)	4 (9.30)	13 (32.50)	15 (55.56)			
Late-pubertal	40 (66.67)	30 (69.77)	25 (62.50)	12 (44.44)			
Post-pubertal	19 (31.67)	9 (20.93)	2 (5.00)	0 (0.00)			
Estimated full-scale IQ	105.88 (10.45)	108.55 (11.84)	105.88 (9.75)	102.87 (12.08)	.92	.11	.11
BMI	23.50 (5.23)	20.72 (3.53)	24.46 (5.62)	21.82 (3.06)	<.001	.17	.92
Smoking (yes/no)	23 (38.33)	2 (4.65)	9 (22.50)	3 (11.11)	<.001	.33	
Sports (hours per week)	3.53 (4.89)	3.73 (2.55)	4.25 (6.19)	6.80 (3.98)	.07	.01	.12
Parental educational status	3.70 (1.03)	4.22 (0.90)	3.36 (0.99)	3.39 (0.76)	.07	<.001	.11
Medication							
Antipsychotics	6 (10.00)	0 (0.00)	0 (0.00)	0 (0.00)	.04	.04	
Stimulants	2 (3.33)	0 (0.00)	1 (2.5)	0 (0.00)	.14	.83	
SSRIs/antidepressants	42 (70.00)	0 (0.00)	28 (70.00)	0 (0.00)	<.001	.90	
Non-stimulants^a^	0 (0.00)	0 (0.00)	1 (2.5)	0 (0.00)	.40	.21	
Tranquilizers	2 (3.33)	0 (0.00)	0 (0.00)	0 (0.00)	.23	.25	
Other^b^	17 (28.33)	5 (11.63)	12 (30.00)	3 (11.11)	<.01	.87	
MDD Severity (DIKJ)	26.85 (9.28)	8.14 (6.30)	22.82 (8.59)	7.22 (4.36)	<.001	.05	.22
Comorbidities							
Lifetime ADHD	4 (6.67)	0 (0.00)	4 (10.00)	0 (0.00)	.02	.53	
Lifetime anxiety disorder	28 (46.67)	2 (4.65)	22 (55.00)	0 (0.00)	<.001	.61	
Lifetime PTSD	7 (11.67)	0 (0.00)	2 (5.00)	1 (3.70)	.04	.53	
Lifetime eating disorder	12 (20.00)	0 (0.00)	1 (2.50)	0 (0.00)	<.01	.02	
Baseline							
Psychological stress	3.20 (3.20)	0.94 (1.62)	2.98 (2.83)	0.80 (1.31)	<.001	.67	.91
HR (bpm)	95.12 (12.07)	95.25 (12.18)	91.77 (11.20)	88.56 (12.52)	.42	.01	.39
RespHRV (msec)	38.86 (17.99)	48.21 (23.96)	38.09 (22.44)	46.41 (19.04)	.01	.70	.88
PEP (msec)	111.60 (12.30)	112.80 (11.05)	109.29 (20.58)	115.97 (9.18)	.08	.85	.22

Data are mean (SD) or n (%). ^a^Non-stimulants include atomoxetine medication. ^b^Other includes usage of asthma medication, painkiller, or vitamin preparation. ADHD=attention deficit and hyperactivity disorder, BMI=body mass index, *bpm *beats per minute, *DIKJ *Children’s Depression Inventory (German Version; for more details see Online Resource 1), *HCs *healthy controls, *HR *heart rate, *MDD *major depressive disorder, *msec *milliseconds, *PEP * pre-ejection period, PTSD=post-traumatic stress disorder, *RespHRV *respiratory heart rate variability, *SSRI *selective serotonin reuptake inhibitor/antidepressant medication

## Correlations of psychological stress and autonomic nervous system measures

Small positive correlations between the AUC_I_ of psychological stress and HR were found in the overall (*n* = 167, *r*=.16, *p*=.04) and MDD subsample (*n* = 99, *r*=.23, *p*=.02), but not in HCs. For RespHRV and PEP measures, no significant correlations with the AUC_I_ of psychological stress in the overall sample nor the HC subsamples were found (*r<.*22, *p≥*.08). While AUC_I_ of RespHRV and AUC_I_ of psychological stress showed a small negative correlation in the MDD group (*n* = 99, *r*=.20, *p*=.05), this did not apply for HCs (*n* = 68, *r*=-.10, *p*=.41).

### RmANCOVAs of psychological and autonomic nervous system stress response

**Fig. 2:**
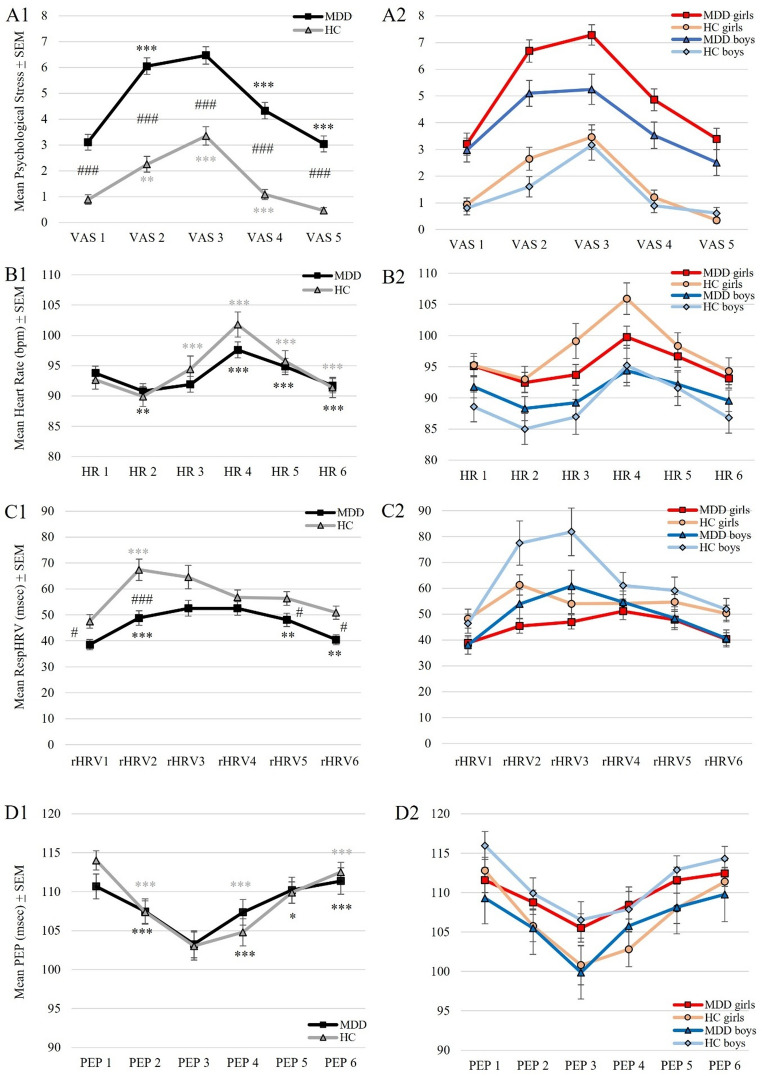
Autonomic and psychological stress response during the Trier Social Stress Test in participants with major depressive disorder (MDD) compared to healthy controls (HCs). *Note*. Psychological Stress (**A**), HR (**B**), RespHRV (**C**), and PEP (**D**) responses to psychosocial stress in the overall (1) and sex-separated groups (2). Significant difference with corresponding previous value: *p < .05, **p < .01, ***p < .001. Significant group difference: #*p* < .05, ##*p* < .01, ###*p* < .001. bpm=beats per minute, HR=heart rate, msec=milliseconds, PEP = pre-ejection period, RespHRV=respiratory heart rate variability

Psychological and ANS stress responses were successfully induced by the TSST in both groups, as indicated by large effects of time for all four dependent variables (Table 2; raw data in Fig. 2 and Table S1a, Table S1b in Online Resource 2). Higher levels of psychological stress (large effect) and lower levels of RespHRV (small effect) were found in the MDD compared to the HC group (main effects of group). The psychological stress reaction over time was greater for the MDD group compared to the HC group [group-by-time interaction effect with *F*(4, 648) = 3.92, *p*=.01, *η²*_*p*_=.02]. In contrast, blunted ANS reactions, i.e., less HR and RespHRV increase as well as less PEP reduction were found in MDD compared to HCs [group-by-time effects: HR, *F*(5, 805) = 3.07, *p*=.03, *η²*_*p*_=.02; RespHRV, *F*(5, 800) = 3.31, *p*=.01, *η²*_*p*_=.02; PEP, *F*(5, 795) = 3.37, *p*=.03, *η²*_*p*_=.02]. No group-by-time-by-sex effect was found for any of the dependent variables. Including an RespHRV measure adjusted for the respiration rate of each time period, its group-by-time effect [*F*(5, 795) = 3.92, *p*<.01, *η²*_*p*_=.02] and group-by-sex-time effect [*F*(5, 795) = 0.03, *p*=.84, *η²*_*p*_<.01] remained unchanged. Data was not fully complete for all analyses (see Online Resource 1), however results did not change when excluding participants with missing data.

**Table 2:** Results of repeated measures analyses of covariance of the psychological and autonomic nervous system stress response in participants with major depressive disorder (MDD) compared to healthy controls (HCs)

	Psychological stress				Heart Rate				RespHRV				PEP			
	df	F	p	η²_p_	df	F	p	η²_p_	df	F	p	η²_p_	df	F	p	η²_p_
Time	4, 648	82.17	<.001	0.34	5, 805	41.13	<.001	0.20	5, 800	27.25	<.001	0.15	5, 795	60.05	<.001	0.28
Group	1, 162	57.28	<.001	0.26	1, 161	<0.01	.97	0.00	1, 160	6.31	.01	0.04	1, 159	0.09	.77	<0.01
Sex	1, 162	1.24	.27	0.01	1, 161	3.04	.08	0.02	1, 160	0.02	.89	<0.01	1, 159	3.76	.05	0.02
Group × time	4, 648	3.92	.01	0.02	5, 805	3.07	.03	0.02	5, 800	3.31	.01	0.02	5, 795	3.37	.03	0.02
Sex x time	4, 648	2.40	.06	0.02	5, 805	2.59	.05	0.02	5, 800	4.34	<.01	0.03	5, 795	2.84	.05	0.02
Group × sex × time	4, 648	1.20	.31	0.01	5, 805	0.80	.50	0.01	5, 800	0.38	.80	<0.01	5, 795	0.68	.53	<0.01

Repeated measures analyses of covariance (rmANCOVA) with group (MDD vs. HCs) and sex (female vs. male) as between-subject factors, and time as within-subject factor. Analyses on autonomic nervous system responses controlled for age, pubertal status, body mass index, smoking, and exercise. Analyses on psychological stress controlled for age and pubertal status. Where necessary, Greenhouse-Geisser corrections were applied, but uncorrected degrees of freedom (df) are reported here for clarity. *PEP *pre-ejection period, *RespHRV *respiratory heart rate variability

### Sensitivity analyses

Including possible effects of comorbid mental disorders and current medication as additional covariates in each rmANCOVA, 94% of the reported effects for all psychological and ANS measures remained unchanged (see Table S2 in Online Resource 2). When including anxiety disorders, group-by-time effects for psychological stress, HR, and PEP were no longer significant (*p*=.06-0.09) although effect sizes remained the same.

Including history or presence of comorbid anxiety disorders/PTSD into the models, the small group-by-time effects for psychological stress, HR, RespHRV, and PEP were still detectable but did not reach significance (except for RespHRV). Therefore, to examine the influence of comorbid anxiety disorders/PTSD more closely, the MDD group was divided into MDD+/-ANX/PTSD and analyzed separately compared to HC-ANX/PTSD. In these comparisons, numerous group-by-time effects no longer reached significance; however, the effect sizes of these comparisons remained comparable both to each other and to the initial full sample results (see Table S4).

Including SSRI/antidepressant medication as additional covariates in each rmANCOVA, no group-by-time effects for psychological stress, HR, and PEP measures were found (*p*=.28-0.50). Subsequently comparing participants with MDD with and without SSRI/antidepressant medication to HCs (see Table S3 and Figure S1 in Online Resource 2), similar group-by-time and group-by-sex-by-time effects for psychological and ANS measures were found compared to rmANCOVAs without considering SSRI/antidepressant medication; only for HR no group-by-time effect emerged (*p*=.10). Bonferroni corrected post-hoc tests for psychological stress response showed no differences between MDD with and without SSRI/antidepressant medication while both differed from HCs for all time points. For RespHRV and PEP measures, MDD with SSRI/antidepressant medication showed less strong stress response compared to MDD without SSRI/antidepressant medication and HCs. Furthermore, we found a significant group difference (MDD+SSRI vs. MDD-SSRI) for MDD severity with MDD+SSRI having more severe MDD symptoms (mean [SD]: MDD+SSRI = 7.86 [1.04], MDD-SSRI=-5.47 [1.03], t(166) = 2,34, *p*<.001). Additionally, all effects remained stable when including psychological stress as a covariate in the analyses on ANS measures (see Table S5).

## Discussion

To the best of our knowledge, this is the largest study to date examining the ANS stress response in a sample of female and male youth with MDD considering separate measures of both the PNS and SNS. HR, RespHRV, and PEP measures were assessed in response to a validated standardized psychosocial stress task in a sample of youth with a current clinical diagnosis of MDD compared to HCs. Our results extend previous research reporting attenuated ANS reactivity in youth with a history of MDD [16, 19].

The TSST induced substantial psychological stress in all participants, supporting effective stress implementation in line with earlier reports [9, 28]. Both female and male youth with MDD showed higher psychological stress at baseline and in response to acute stress compared to HCs. This is consistent with earlier findings [39], indicating youth with MDD to be more psychologically vulnerable for stronger subjective stress experience. Furthermore, the psychological stress response only correlated with ANS measures in the MDD group and not in the HC group, suggesting group-specific differences in ANS responses despite psychological stress reaction over time for both groups.

For ANS measures, all assessed physiological variables (HR, RespHRV, PEP) reacted to the TSST in youths with MDD as well as HCs, comparable to previous reports [9, 28]. Our study is the first to investigate HR response to acute psychosocial stress in youth with MDD compared to HCs. As expected, youth with MDD showed blunted HR responses to stress, extending adult findings [10] to a population of youth. Regarding the PNS, youth with MDD showed blunted RespHRV stress reactivity compared to HCs in the main analysis and when using the respiration adjusted RespHRV measure. These findings replicate previous studies in youth with a history of MDD [19] or adults with MDD [10]. Moreover, our results indicate impaired parasympathetic functioning in youth with MDD not only in response to stress but also at rest. This parasympathetic alteration is consistent with earlier findings in youth and adults with MDD [10, 19, 40], and may represent a state of general alertness in individuals with MDD. While previous work has primarily examined the PNS, our study extends prior research by investigating the SNS stress response in youth with MDD. Our findings indicate reduced PEP decrease in girls and boys with MDD compared to HCs during psychosocial stress, which is in line with previous work in youth [16] and adults [20]. Hence, individuals with MDD seem to experience blunted SNS reactivity to acute psychosocial stress. On the contrary, our findings in HCs follow previously reported SNS increases in response to psychosocial stress in healthy youth [9] supporting SNS increase as a substantial part of a healthy human stress response. Thus, youth with MDD may be physiologically restricted in their functional SNS response to acute stress. However, descriptively, data suggest these findings to apply only to girls with boys’ data showing a different pattern (SNS hyperactivity), yet this effect was not supported by statistical analyses. Therefore, further studies with larger sample sizes examining potential sex differences are needed to replicate our findings.

We did not find sex to influence the psychophysiological stress reactivity across groups. While previous results (applying different stress procedures and rarely accounting for potential confounders) using a Go/No-Go Task suggest boys with MDD to experience a more severely altered ANS reactivity than girls [18], we found that female and male youth with MDD did not differ in blunted PNS, SNS, or HR reactivity, possibly suggesting an equally altered ANS stress reactivity between sexes. Heterogeneity of the applied stressor tasks might account for diverging results also with regard to previous reports of females being more prone for social stressors and males more sensitive to achievement challenges [41].

The reported blunted ANS stress response suggests a general ANS hypo-reactivity in youth with MDD during acute psychosocial stress. Since ANS hypo-reactivity was still evident when controlling for the psychological stress response we prove the stability of the results and clarify that ANS measures not only reflect clinical state or stress but rather a unique physiological stress response. This is also supported by research considering the TSST as being the gold-standard for the investigation of the cognitive neurobiology of acute stress which has already evidenced different stress responses in various populations (e.g., HC, MDD, ANX) [28].

Altered sympathetic stress responsivity may impair successful stress regulation as affected individuals might not physiologically respond adequately to external or internal threats [7]. The attenuated ANS response might reflect a reduced ability to adapt physiologically to situational demands, which aligns with previous research indicating impaired stress regulation abilities in youth with MDD [42]. Since ANS function maturates during childhood and youth [5], and early life stress is a major risk factor for MDD onset in youth [43], ANS stress reactivity might already be impaired before MDD onset. It therefore remains unclear if ANS dysregulation serves as a vulnerability increasing risk for MDD, or vice versa, follows MDD onset. Thus, future, at best longitudinal studies are needed to investigate developmental trajectories of stress regulation alterations in youths with MDD, including the role of early life stress and its effects on MDD development.

Taken together, stress regulation may play a crucial role in the emergence and maintenance of MDD. Considering the reported ANS alterations, appropriate treatments supporting successful stress management in individuals affected by MDD are warranted. One possibility may be PNS, e.g. vagal, stimulation. Indeed, there is evidence supporting the effectiveness of vagal stimulation in MDD showing mood-enhancing effects in MDD [44]. While only few studies exist in this field, particularly in youth, research on transcutaneous vagus nerve stimulation (tVNS) has gained interest because of the potential of modulating parasympathetic effects on depressive symptoms [45]. If parasympathetic hypoactivity is supported by further studies as a characteristic feature of youth with MDD, further investigation of tVNS in youth would be particularly promising, as it offers a non-invasive alternative to conventional VNS. Biofeedback techniques may also support individuals with MDD in learning to observe and influence physiological reactions to more adequately adjust to stressful situations [46]. Most previous research has focused on the PNS; however, one study reported a decrease in otherwise elevated muscle sympathetic nerve activity during stress in MDD patients following antidepressant treatment, coinciding with symptom improvement [47]. It remains unclear whether sympathetic modulation directly contributes to therapeutic efficacy, yet these findings might also be relevant for future antidepressant medical therapies. However, this has yet to be studied in youth with MDD.

This study has several strengths. We included a large and representative sample of female and male youth with MDD and HCs with comparable sex, age, and pubertal status supporting the investigation of sex effects. We involved a large clinical sample of youth with current clinical diagnosis of MDD (contrary to previous ANS work focusing on youth with a history of MDD [19] or high-risk populations [48]). Participants with MDD and HCs were reliably diagnosed using standardized semi-structured diagnostic interviews based on DSM-IV-TR criteria. As acute stress paradigm, we applied the widely used and validated TSST [8] which proved effective given that both groups experienced increased psychological stress. We also substantially extend previous work by investigating the parasympathetic as well as sympathetic ANS, next to a combined parameter to allow a universal understanding of ANS functioning. ECG and ICG data were controlled and edited under blinded conditions to prevent interpretation bias. Adherence to all procedures (e.g., TSST, ANS measurement) was ensured by standard operating procedures controlled by regular internal monitoring. Finally, we included relevant confounders (e.g., age, pubertal status, BMI, exercise, smoking) in statistical analyses, and confirmed the specificity and stability of our results in additional sensitivity analyses.

Nevertheless, our study also has some limitations. First, our sample comprised a higher rate of female compared to male participants given the higher prevalence of MDD in girls than boys [2]. However, sex was equally distributed in the MDD and HC group. Male participants tended to have lower pubertal status than female participants, in line with earlier pubertal onset in females than males. Thus, all analyses were controlled for pubertal status. Moreover, our sample did not include children under the age of 12 because of the lower prevalence of MDD compared to youth [1]. In addition, selecting participants in an at least mid-pubertal stage (given low numbers of pre-pubertal cases with MDD) our current work including analyses of possible sex differences is limited to pubertal adolescents with MDD. Future studies should therefore investigate ANS stress response and its possible sex differences in pre-pubertal children with MDD. Second, exercise as well as alcohol and substance use on the day of assessment was only acquired via self-report. As recent findings show only low-to-moderate agreement between self-reports and biospecimens [49] future research should include valid questionnaires or testing procedures to allow objective verification. Third, correlational analyses between the four variables relied solely on measures of AUC with respect to increase and should additionally be backed up by reactivity or residualized scores in future research. Fourth, PNS was only operationalized via RSA while other markers such as the “Root mean square of successive differences“ (RMSSD) may be more robust to respiratory influences and therefore provide a more accurate representation of the PNS [50]. However, evidence is not consistent as other research finds RSA preferable compared to RMSSD [51]. Fifth, as our MDD sample covers predominantly patients recruited from the clinic, they showed high rates of SSRI/antidepressant medication which has been reported to influence the ANS [28, 52]. However, comparing ANS reactivity between medicated and unmedicated participants with MDD to HCs, group differences for both the PNS and SNS were still evident. Medicated participants had more severe depressive symptoms than unmedicated participants, leading to the consideration that blunted ANS response for medicated compared to unmedicated participants might be due to stronger illness severity. Future studies should include larger unmedicated samples and include illness severity to diminish possible medication effects. Sixth, our clinical sample included participants with various comorbid mental disorders which werenot evenly distributed between groups. Also, HCs were not free of lifetime mental disorders, yet in much lower rates. To ensure the specificity of our results for youth with MDD, we controlled for comorbid mental disorders in additional sensitivity analyses. Although most effects remained stable, the number of participants affected by comorbid mental disorders might not have been large enough to detect effects of psychiatric comorbidity. Moreover, comorbid anxiety disorders altered the significance of the main effects of psychological stress, HR and PEP, suggesting an influence of anxiety on ANS reactivity. Consequently, it must be considered whether the findings are disorder-specific or reflect patterns common across affective disorders. Existing evidence regarding ANS responses in children with anxiety disorders, however, suggest individual ANS patterns for anxiety disorders which differ from our results in youth [53]. Furthermore, we found no difference in effect size between youth with MDD with and without anxiety disorders compared to healthy youth. Future work focusing on characteristics of clinically relevant subtypes should include larger samples of participants with comorbid mental disorders.

## Conclusion

This work extends previous work by providing evidence of an altered ANS stress response of both the PNS and SNS in female and male youth with MDD compared to HCs. Blunted ANS reactivity was found together with increased subjective psychological stress, suggesting a maladaptive psychophysiological regulation of stressful situations. Considering physiological alterations in youth with MDD may help to improve future clinical interventions and individual treatments.

## Supplementary Information

Below is the link to the electronic supplementary material.

Supplementary Material 1

## Data Availability

Data of this study are not openly available as participants, or their parents did not give consent for their data to become fully open to the scientific community. However, interested researchers can apply for data access as part of a collaboration.

## Code Availability

Codes of statistical analyses can be made available upon reasonable request.
