# Supplementary material for: Stress reactivity of the autonomic nervous system in youth with and without major depressive disorder
Source: Eur Child Adolesc Psychiatry. 2026 Mar 25;35(7):2189–200. doi: 10.1007/s00787-026-02998-5 (PMC13427802; doi:10.1007/s00787-026-02998-5)
Supplement: Supplementary file 1 — Supplementary Material 1 [file 787_2026_2998_MOESM1_ESM.docx]

**Supplementary Material**

**Stress Reactivity of the Autonomic Nervous System in Youth With and Without Major Depressive Disorder**

*Running head*: ANS Stress Reactivity in Youth with MDD

*Journal:* European Child & Adolescent Psychiatry

Nikola Fann^1^, Anne Martinelli^1,2 (0000-0002-7158-9778)^, Helena Oldenhof^3 (0000-0002-7210-1519)^, Christine M. Freitag^1 (0000-0001-9676-4782)^, & Anka Bernhard^1,4^ ^(0000-0001-8864-1360)^

^1^Department of Child and Adolescent Psychiatry, Psychosomatics and Psychotherapy, University Hospital Frankfurt am Main, Goethe University, Frankfurt am Main, Germany

^2^Fresenius University of Applied Sciences Frankfurt am Main, Frankfurt am Main, Germany

^3^Amsterdam UMC, Vrije Universiteit Amsterdam, The Netherlands

^4^Department of Child and Adolescent Psychiatry and Psychotherapy, Faculty of Medicine, Technische Universität Dresden, German Center for Child and Adolescent Health (DZKJ), partner site Leipzig/Dresden, Dresden, Germany

*Corresponding author:*

Anka Bernhard, PhD, Department of Child and Adolescent Psychiatry and Psychotherapy, Faculty of Medicine, Technische Universität Dresden, German Center for Child and Adolescent Health (DZKJ), partner site Leipzig/Dresden, Dresden, Germany, Fetscherstraße 74, D-01307 Dresden, Germany; e-mail: anka.bernhard@ukdd.de; phone: +49351458118912

**Online Resource 1**

**Additional Information to Procedures**

**Psychiatric disorders**

Current and lifetime psychiatric disorders were assessed with the Schedule for Affective Disorders and Schizophrenia for School-Age Children – Present and Lifetime Version (K-SADS-PL) [1] completed with youth and their caretaker separately by trained staff. All staff were postgraduate students of psychology trained by experienced clinical psychologists. Interviewers completed a minimum of three supervised interview sessions with at least 90% concordance in score ratings, following the gold-standard method for interviewer training [2]. The KSADS-PL is a standardized semi-structured interview for assessing current and past mental disorders according to DSM-IV-TR criteria. DSM-IV-TR diagnoses were based on information of both parent and youth. According to the K-SADS-PL instructions the interviewer followed their best clinical judgment in assigning the summary ratings including all sources of information (e.g., youth, parent, school, clinic), particularly in case of discrepancies between sources. The most frequent disagreements occurred for items related to subjective phenomena (e.g., the youth described the presence or absence of certain symptoms, of which the parent wasn’t informed of or hadn’t observed, such as items of guilt, hopelessness, sleep disturbances, hallucinations, or suicidal ideation). If the disagreements were related to observable behaviors (e.g., truancy, fire setting, compulsive ritual), the interviewer queried sources about the discrepant information. Finally, the interviewer was advised to use his or her best clinical judgment in assigning the summary ratings. If needed, the case would have also been discussed with experienced clinicians within the research team. The K-SADS-PL is a valid and reliable instrument with interrater agreement of 98% across all diagnoses and a test-retest reliability between 0.55 and 1.00, with highest values for Major Depressive Disorder (Cohen’s κ=.90–1.00) [1].

**IQ**

To estimate IQ based on a verbal and a non-verbal subtest, the vocabulary test and matrix reasoning test of the Wechsler Intelligence Scale-IV were used. If the participants were <16 years old, the German version of the Wechsler Intelligence Scale for Children (WISC) [3] - the Hamburg-Wechsler-Intelligence-test for children IV (HAWIK-IV) - was applied. If participants were >16 years old, the Wechsler Intelligence-test for adults (WIE) - the German version of the Wechsler Adult Intelligence Scale (WAIS) [4] - was performed. Estimated IQ was defined as the arithmetic mean of the scored IQ-points of the two subscales.

**Socioeconomic status**

Parental educational status was investigated as a proxy for parental socioeconomic status. The International Standard Classification of Education (ISCED) criteria [5] were assessed independently for both caretakers, and the mean of both caretakers or the value of one caretaker in case of missing information of the other caretaker was taken as level of education. ISCED allows rating of the highest educational status on a six-categorical scale from 0=pre-primary level of education, 1=primary level of education, 2=lower secondary level of education, 3=upper secondary level of education, 4=post-secondary level of education, 5=first stage of tertiary education, to 6=second stage of tertiary education.

**Pubertal development**

The Pubertal Development Scale (PDS) [6] is a self-report measure of pubertal status. It consists of five items on pubertal growth related to body hair change, combined with voice and facial hair change in males, and breast development and menarche in females. Scoring options for the items range on a four-level scale between 0=”not yet” to 3=”seems complete”, resulting in a five-level categorial variable (0=pre- /1=early- /2=mid- /3=late- /4=post-pubertal) drawn from the sum score. The PDS reveals high validity and good internal consistency with an α coefficient median of .77 [6]. Validity is supported by moderate to high correlations with the clinician-rated Tanner stage [7].

**MDD severity**

MDD severity was assessed by the self-report questionnaire “Depressionsinventar für Kinder und Jugendliche” (DIKJ; [8]), the German version of the Children’s Depression Inventory (CDI) [9]. The DIKJ consists of 26 items about the severity or frequency of depressive symptoms on a three-level scale between 0 and 2 (e.g., 0=“I feel exhausted seldomly”, 1=“I often feel exhausted”, 2=“I always feel exhausted”). It shows high internal consistency with an α coefficient between .87 and .92 [8].

**Body mass index**

Current body mass index (BMI; weight[kg]/height[m]²) of participants were calculated using measured weight and height at assessment time.

**Smoking**

Current smoking of nicotine (cigarettes/day) was assessed via self-report of participants on the number of daily smoked cigarettes (“How many cigarettes do you smoke on an average day?”) and transferred into a nominal variable (smoking yes/no) for analyses.

**Exercise**

Current engagement in sports was assessed via self-report of participants on the hours of regular engagement in sports per week (“How many hours per week do you engage in exercising?”).

**Medication**

Current medication use (substance name, time of intake, dose) was assessed via self-report of participants about their regularly taken medication and on the day of assessment, and if reported, categorized as follows: antipsychotics (neuroleptics), stimulants (methylphenidate, amphetamine), non-stimulants (atomoxetine, guanfacine), selective serotonin reuptake inhibitor/antidepressants, tranquilizers (benzodiazepines), or other (e.g., vitamins, iron, pain killers, allergy/asthma medication).

**Additional Information to the Trier Social Stress Test**

The Trier Social Stress Test (TSST) is a reliable and valid method to induce psychosocial stress [10, 11]. It is widely used in psychophysiological research and considered to be the gold standard of acute stress assessment in laboratory conditions [12]. A child-adapted version of the TSST was used [13]. The TSST appointment was scheduled in the afternoon to minimize circadian influences on the measurements (starting time around 2pm). If possible, TSST appointments for female youth were scheduled during the luteal phase of the menstruation cycle to ensure better comparison with male participants [14]. Alcohol/substance use was not permitted on the assessment day nor drinking, eating, or smoking during the whole experiment. Before starting the TSST, participants were asked for possible substance use, caffeine consumption, and exercise prior to the session. If participants affirmed to substance use, the TSST session was re-scheduled. Caffeine consumption or exercise prior to the session were tolerated because of an implemented 60-minute relaxation period before the beginning of the TSST. In a comfortable room (Room A) the ANS measurement hardware was connected whereupon an approximately 50 minute period of sitting down and adjusting to the connected hardware followed. Afterwards the first ten-minute resting period in a standing position started. Subsequently participants were accompanied down the hall to a less comfortable room (Room B), located approximately three meters from Room A, without stairs. Here they were instructed that they had to give a five-minute speech in front of a panel of two strangers (judges did not wear lab coats) about a favorite book, movie or TV series including the storyline and their opinions of the characters and plot. Participants were told their performance was going to be filmed and analyzed later for comparison purposes, therefore they should try to deliver a superior performance compared to the other participants. However, no video recording was done and participants debriefed directly at the end of the TSST. Before the speech task, participants had three minutes preparation time to plan their speech. They were able to make notes which they were not allowed to use during the speech. During the speech task the two unfamiliar jury members were instructed to appear emotionally neutral and to instruct the participants to go on with their speech if it had not yet lasted five minutes. After five minutes the speech task was finished, and the children stopped irrespective whether they had finished their speech. Next, the five-minute mental arrhythmic task was explained by the jury. According to the TSST adaptation for children [13], participants were instructed to serially subtract a fixed number from a given starting number (for participants aged 12 years and older: 13 from 1023) as fast and accurately as possible. In case of a wrong answer, participants were instructed to start from the beginning. After the speech and mental arithmetic task, participants left Room B and re-entered the more comfortable Room A where they received positive feedback for their performance and were fully debriefed followed by another ten-minute recovery period in standing position. To avoid postural effects on cardiac measurements, participants remained in a standing position from the beginning of the initial resting period until the end of the post resting period. To confirm psychological stress induction, participants rated their feelings of stress (“Do you feel stressed?”) after the pre-task standing period (1), the explanation of the speech task (2), the public speech task (3), the mental arithmetic task (4), and the post-task standing period (5) using a Visual Analogue Scale (VAS) ranging between 0 and 10 (0=no, not at all to 10=yes, very much) [15].

**Additional Information to the Autonomic Nervous System Measurement**

*VU-AMS hard- and software*: The VU-AMS (Vrije Universiteit Ambulatory Monitoring System) is a valid ambulatory device recording electrocardiography (ECG) and impedance cardiography (ICG) data [16, 17]. The sampling frequency was set at 1000 Hz. Signals were derived using seven micropore electrodes positioned (1) under the right clavicular bone 4 cm right of the sternum (ECG V-), (2) at the apex of the heart over the ninth rib on the left lateral margin of the chest, approximately at the level of the processus xiphoideus (ECG V+), (3) on the right side, between the lower two ribs (Ground), (4) at the suprasternal notch above the top of the sternum (ICG V-), (5) at the processus xiphoideus at the bottom of the sternum (ICG V+), (6) at the back on the spine at least 3 cm above electrode ICG V- (ICG I-), and (7) at the back on the spine at least 3 cm below electrode ICG V+ (ICG I+) [18]. The VU-DAMS 4.3 software package was used for generating the raw data output of the recorded ECG and ICG. The VU-DAMS 4.3 software used an ensemble averaging calculation. The distance between ICG electrodes was measured and considered because of its influence on thorax impedance. ECG quality was manually checked by the first author under blinded conditions. Noise signals, supraventricular and ventricular extrasystoles were marked and excluded from the analysis. Participants with incomplete data, >50% discarded data in single time-periods, and participants completely missing ECG and ICG measurements were excluded. After manual setting of time-periods of each participant file (VU-DAMS 4.3=“labels”), the implemented algorithms identified R-peaks, Q-wave onsets, B-points, and X-points for each individual time-period during the TSST [19]. R-peaks, Q-waves, B-points, and X-points were also manually checked, and if needed corrected under blinded conditions according to the Data Analysis and Management Software (DAMS) of the VU-AMS Manual version 1.5 [18] and previous work [20, 21]. If necessary, a second check by a blinded and trained researcher was done. HR was calculated through the R-peak time series of the ECG and measured in beats per minute (bpm). RSA (RespHRV) scores were derived from the recorded ECG and respiration data as follows: the shortest interbeat interval during inspiration was subtracted from the longest interbeat interval during expiration [16]. This time-based method calculating RSA (RespHRV) values is known to be equivalent to other methods measuring HRV [22]. PEP is defined as the time between the beginning of ventricular depolarization marked by the Q-wave onset in the ECG and the opening of the aortic valve marked by the B-point in the ICG [20, 23]. The VU-AMS software uses the R-wave as an approximation for the beginning of the systole as this method is known to be more reliable than using the Q-wave onset [19, 24]. HR, RSA (RespHRV) and PEP were computed as mean values across each defined time period. Higher RSA (RespHRV) values in milliseconds (msec) indicate higher parasympathetic activity, whereas higher PEP values (msec) indicate lower sympathetic PEP values.

**Additional Information to Statistical Analyses**

Data was not complete for all measures, as there was missing psychological stress data of one female with MDD and one HC male leading to their exclusion in the rmANCOVA with psychological stress as dependent variable. For the RSA analysis data of one HC male was missing. For the PEP analysis, data of one HC female was missing, and data of one MDD male was excluded due to being an outlier. HR data and data of all covariates were complete for all analyses. For clarity, full group sizes are reported. Descriptive data regarding significant group or sex differences did not change when excluding the five participants with missing data.

**Online Resource 2**

**Supplementary Tables and Figures**

| **Table S1a.** Original data of the autonomic nervous system and psychological stress in response to the Trier Social Stress Task in participants with major depressive disorder (MDD) compared to healthy controls (HCs). | | | | |
| --- | --- | --- | --- | --- |
|  | Females (*n*=103) | | Males (*n*=67) | |
|  | MDD (*n*=60) | HCs (*n*=43) | MDD (*n*=40) | HCs (*n*=27) |
| Heart rate (bpm) |  |  |  |  |
| Baseline standing | 95.12 (12.07) | 95.25 (12.18) | 91.77 (11.20) | 88.56 (12.52) |
| Task introduction | 92.45 (12.55) | 92.98 (13.48) | 88.28 (12.13) | 85.02 (13.04) |
| Task preparation | 93.69 (12.91) | 99.12 (18.46) | 89.23 (12.83) | 86.94 (14.66) |
| Speech task | 99.76 (13.54) | 105.93 (16.49) | 94.39 (12.05) | 95.20 (16.93) |
| Arithmetic task | 96.66 (13.58) | 98.32 (14.20) | 92.18 (12.44) | 91.58 (14.60) |
| Recovery standing | 93.13 (12.05) | 94.27 (14.07) | 89.55 (10.89) | 86.81 (12.76) |
| AUC_I_ | 1.07 (29.69) | 14.86 (45.03) | -4.09 (23.06) | 3.60 (47.73) |
| RespHRV (msec) |  |  |  |  |
| Baseline standing | 38.86 (17.99) | 48.21 (23.96) | 38.09 (22.44) | 46.41 (19.04) |
| Task introduction | 45.40 (21.08) | 61.33 (25.65) | 53.93 (35.02) | 77.46 (43.54) |
| Task preparation | 46.96 (21.02) | 54.05 (25.18) | 60.87 (39.23) | 81.88 (46.84) |
| Speech task | 51.14 (25.35) | 54.19 (21.59) | 54.60 (27.68) | 61.05 (26.42) |
| Arithmetic task | 47.85 (24.45) | 54.67 (19.36) | 48.41 (28.03) | 59.17 (26.72) |
| Recovery standing | 40.32 (19.11) | 50.29 (20.91) | 40.59 (21.15) | 51.86 (21.70) |
| AUC_I_ | 36.66 (59.89) | 32.45 (90.54) | 66.68 (64.04) | 96.66 (123.42) |
| PEP (msec) |  |  |  |  |
| Baseline standing | 111.60 (12.30) | 112.80 (11.05) | 109.29 (20.58) | 115.97 (9.18) |
| Task introduction | 108.78 (12.00) | 105.79 (13.20) | 105.50 (21.03) | 109.92 (10.26) |
| Task preparation | 105.53 (13.88) | 100.80 (16.51) | 99.87 (21.40) | 106.54 (12.00) |
| Speech task | 108.41 (13.62) | 102.81 (14.18) | 105.78 (19.81) | 107.90 (14.57) |
| Arithmetic task | 111.59 (12.77) | 108.01 (12.54) | 108.14 (21.31) | 112.88 (9.34) |
| Recovery standing | 112.45 (12.68) | 111.40 (11.48) | 109.77 (21.77) | 114.33 (8.13) |
| AUC_I_ | -11.64 (17.26) | -35.05 (34.57) | -17.63 (20.99) | -27.47 (28.07) |
| Psychological stress (range 1-10) | |  |  |  |
| VAS 1 | 3.20 (3.20) | 0.94 (1.62) | 2.98 (2.83) | 0.80 (1.31) |
| VAS 2 | 6.69 (3.25) | 2.65 (2.79) | 5.10 (3.05) | 1.60 (1.97) |
| VAS 3 | 7.28 (2.96) | 3.46 (2.96) | 5.25 (3.59) | 3.16 (2.30) |
| VAS 4 | 4.86 (3.13) | 1.21 (1.79) | 3.53 (3.13) | 0.89 (1.34) |
| VAS 5 | 3.40 (3.08) | 0.35 (0.69) | 2.51 (3.05) | 0.61 (1.19) |
| AUC_I_ | 9.26 (8.72) | 4.22 (6.35) | 4.71 (8.08) | 3.22 (3.48) |
| *Note*: All data are displayed as mean (SD). AUC_I_=area under the curve with respect to increase, Bpm=beats per minute, HCs=healthy controls, MDD=major depressive disorder, msec=milliseconds, PEP=pre-ejection period, RespHRV =respiratory heart rate variability, VAS=visual analogue scale. | | | | |

| **Table S1b.** Mean differences with 95% confidence intervals (CI) of the autonomic nervous system and psychological stress in response to the Trier Social Stress Task in participants with major depressive disorder (MDD) compared to healthy controls (HCs). | | | | |
| --- | --- | --- | --- | --- |
|  | Mean Difference | Standard Error | 95% CI, Lower Bound | 95% CI, Upper Bound |
| Heart rate (bpm) |  |  |  |  |
| Baseline standing | 1.87 | 1.94 | -1.96 | 5.70 |
| Task introduction | 1.19 | 2.15 | -3.05 | 5.44 |
| Task preparation | -1.48 | 2.53 | -6.48 | 3.53 |
| Speech task | -2.60 | 2.50 | -7.54 | 2.35 |
| Arithmetic task | .24 | 2.28 | -4.26 | 4.75 |
| Recovery standing | 1.31 | 1.99 | -2.62 | 5.23 |
| RespHRV (msec) |  |  |  |  |
| Baseline standing | -.20* | .09 | -.37 | -.03 |
| Task introduction | -.34** | .08 | -.50 | -.17 |
| Task preparation | -.13 | .10 | -.31 | .06 |
| Speech task | -.07 | .09 | -.24 | .10 |
| Arithmetic task | -.18* | .09 | -.36 | -.01 |
| Recovery standing | -.22* | .08 | -.38 | -.05 |
| PEP (msec) |  |  |  |  |
| Baseline standing | .11 | .07 | -.02 | .24 |
| Task introduction | .01 | .06 | -.10 | .13 |
| Task preparation | .05 | .07 | -.09 | .18 |
| Speech task | -.08 | .10 | -.28 | .12 |
| Arithmetic task | -.01 | .08 | -.17 | .14 |
| Recovery standing | .03 | .06 | -.08 | .14 |
| Psychological stress (range 1-10) | |  |  |  |
| VAS 1 | 2.09** | .42 | 1.27 | 2.91 |
| VAS 2 | 3.55** | .47 | 2.63 | 4.48 |
| VAS 3 | 2.62** | .51 | 1.63 | 3.62 |
| VAS 4 | 2.99** | .43 | 2.13 | 3.84 |
| VAS 5 | 2.25** | .40 | 1.47 | 3.03 |
| *Note*: Bpm=beats per minute, msec=milliseconds, PEP=pre-ejection period, RespHRV =respiratory heart rate variability, VAS=visual analogue scale, **=*p*<.001, *=*p*<.05. | | | | |

**Table S2**. Results of repeated measures analyses of co-variance of the psychological and autonomic nervous system stress response in participants with major depressive disorder (MDD) compared to healthy controls (HCs) considering additional potentially confounding effects.

|  | *df* | *F* | *p* | *η²_p_* | ADHD | ANX | PTSD | EAT | APSY | SSRI | OTHER |
| --- | --- | --- | --- | --- | --- | --- | --- | --- | --- | --- | --- |
| PSYCH. STRESS |  |  |  |  |  |  |  |  |  |  |  |
| Time | 4, 648 | 82.17 | **<.001** | 0.34 | yes | yes | yes | yes | yes | yes | yes |
| Group | 1, 162 | 57.28 | **<.001** | 0.26 | yes | yes | yes | yes | yes | yes | yes |
| Sex | 1, 162 | 1.24 | .27 | 0.01 | yes | yes | yes | yes | yes | yes | yes |
| Group x time | 4, 648 | 3.92 | **.01** | 0.02 | yes | .09 | yes | yes | yes | .28 | yes |
| Sex x time | 4, 648 | 2.40 | .06 | 0.02 | yes | yes | yes | .03 | .04 | yes | yes |
| Group x sex x time | 4, 648 | 1.20 | .31 | 0.01 | yes | yes | yes | yes | yes | yes | yes |
| HEART RATE |  |  |  |  |  |  |  |  |  |  |  |
| Time | 5, 805 | 41.13 | **<.001** | 0.20 | yes | yes | yes | yes | yes | yes | yes |
| Group | 1, 161 | <0.01 | .97 | 0.00 | yes | yes | yes | yes | yes | yes | yes |
| Sex | 1, 161 | 3.04 | .08 | 0.02 | yes | yes | yes | yes | yes | yes | yes |
| Group x time | 5, 805 | 3.07 | **.03** | 0.02 | yes | .06 | yes | yes | yes | .28 | yes |
| Sex x time | 5, 805 | 2.59 | .05 | 0.02 | yes | yes | yes | yes | yes | yes | yes |
| Group x sex x time | 5, 805 | 0.80 | .50 | 0.01 | yes | yes | yes | yes | yes | yes | yes |
| RespHRV |  |  |  |  |  |  |  |  |  |  |  |
| Time | 5, 800 | 27.25 | **<.001** | 0.15 | yes | yes | yes | yes | yes | yes | yes |
| Group | 1, 160 | 6.31 | **.01** | 0.04 | yes | yes | yes | yes | yes | .50 | yes |
| Sex | 1, 160 | 0.02 | .89 | <0.01 | yes | yes | yes | yes | yes | yes | yes |
| Group x time | 5, 800 | 3.31 | **.01** | 0.02 | yes | yes | yes | yes | yes | yes | yes |
| Sex x time | 5, 800 | 4.34 | **<.01** | 0.03 | yes | yes | yes | yes | yes | yes | yes |
| Group x sex x time | 5, 800 | 0.38 | .80 | <0.01 | yes | yes | yes | yes | yes | yes | yes |
| PEP |  |  |  |  |  |  |  |  |  |  |  |
| Time | 5, 795 | 60.05 | **<.001** | 0.28 | yes | yes | yes | yes | yes | yes | yes |
| Group | 1, 159 | 0.09 | .77 | <0.01 | yes | yes | yes | yes | yes | yes | yes |
| Sex | 1, 159 | 3.76 | .05 | 0.02 | yes | yes | .02 | yes | yes | yes | yes |
| Group x time | 5, 795 | 3.37 | **.03** | 0.02 | yes | .09 | yes | yes | yes | .38 | yes |
| Sex x time | 5, 795 | 2.84 | .05 | 0.02 | yes | yes | yes | yes | yes | yes | yes |
| Group x sex x time | 5, 795 | 0.68 | .53 | <0.01 | yes | yes | yes | yes | yes | yes | yes |

*Note.* Repeated measures analyses of co-variance (rmANCOVA) with group (MDD vs. HCs) and sex (female vs. male) as between-subjects factors, and time as a within-subject factor, respectively, controlled for major confounders (psychological stress: age, pubertal status; neuroendocrine measures: age, pubertal status, body mass index, smoking, exercise). Where necessary, Greenhouse Geisser corrections were applied, but uncorrected degrees of freedom (df) are reported here for clarity. Columns of sensitivity analysis indicate whether effects hold the same significance level *p*<.05 vs. *p*>.05 (yes; if not, *p*-value is displayed) as in the respective rmANCOVA without the additional covariate. ADHD=attention-deficit/hyperactivity disorder, ANX=anxiety disorders, APSY=Antipsychotic medication, EAT=eating disorders, Other includes usage of asthma medication, painkiller, or vitamin preparation, PEP=pre-ejection period, PTSD=posttraumatic stress disorder, RespHRV=respiratory heart rate variability, SSRI=selective serotonin reuptake inhibitor/antidepressant medication. *η²_p_*=partial eta squared.

| **Table S3.** Results of repeated measures analyses of covariance (rmANCOVA) of the psychological and autonomic nervous system stress response in youth with major depressive disorder with (*n*=70) and without (*n*=30) current serotonin-reuptake-inhibitor (SSRI)/antidepressant medication compared to healthy controls (*n*=70). | | | | | | | | | | | | | | | | |
| --- | --- | --- | --- | --- | --- | --- | --- | --- | --- | --- | --- | --- | --- | --- | --- | --- |
|  | Psychological stress | | | | Heart Rate | | | | RespHRV | | | | PEP | | | |
|  | *df* | *F* | *p* | *η²_p_* | *df* | *F* | *p* | *η²_p_* | *df* | *F* | *p* | *η²_p_* | *df* | *F* | *p* | *η²_p_* |
| Time | 4, 640 | 77.72 | **<.001** | 0.33 | 5, 795 | 29.51 | **<.001** | 0.16 | 5, 790 | 27.56 | **<.001** | 0.15 | 5, 785 | 47.34 | **<.001** | 0.23 |
| Group | 2,160 | 30.29 | **<.001** | 0.28 | 2,159 | 0.01 | .99 | <.001 | 2, 158 | 4.50 | **.01** | 0.05 | 2, 157 | 2.90 | .06 | 0.04 |
| Sex | 1,160 | 1.24 | .27 | 0.01 | 1,159 | 0.84 | .36 | 0.01 | 1, 158 | 0.08 | .78 | <0.01 | 1, 157 | 1.08 | .30 | 0.01 |
| Group x time | 8, 640 | 2.18 | **.04** | 0.03 | 10, 795 | 1.81 | .10 | 0.02 | 10, 790 | 3.15 | **<.01** | 0.04 | 10, 785 | 3.63 | **<.01** | 0.04 |
| Sex x time | 4, 640 | 2.40 | .08 | 0.01 | 5, 795 | 2.79 | **.04** | 0.02 | 5, 790 | 5.53 | **<.001** | 0.03 | 5, 785 | 2.37 | .08 | 0.02 |
| Group x sex x time | 8, 640 | 1.20 | .68 | <0.01 | 10, 795 | 1.09 | .37 | 0.01 | 10, 790 | 1.80 | .08 | 0.02 | 10, 785 | 0.89 | .49 | 0.01 |
| *Note*: Repeated measures analyses of covariance (rmANCOVA) with group (MDD+SSRI vs. MDD-SSRI vs. HCs) and sex (female vs. male) as between-subjects factors, and time as within-subject factor. Autonomic nervous system responses controlled for age, pubertal status, body mass index, smoking, and exercise, while psychological stress measure controlled for age and pubertal status. Where necessary, Greenhouse-Geisser corrections were applied, but uncorrected degrees of freedom (df) are reported here for clarity. PEP=pre-ejection period, RespHRV=respiratory heart rate variability. | | | | | | | | | | | | | | | | |

| **Table S4**. Results of repeated measures analyses of co-variance (rmANCOVA) of the psychological and autonomic nervous system stress response in participants with major depressive disorder (MDD) with (+) and without (-) lifetime anxiety disorder and/or PTSD compared to healthy controls (HCs) without lifetime anxiety disorder and/or PTSD. | | | | | | | | | | | | | | | | |  |
| --- | --- | --- | --- | --- | --- | --- | --- | --- | --- | --- | --- | --- | --- | --- | --- | --- | --- |
|  | Psychological stress | | | | Heart Rate | | | | RespHRV | | | | PEP | | | | |
|  | *df* | *F* | *p* | *η²_p_* | *df* | *F* | *p* | *η²_p_* | *df* | *F* | *p* | *η²_p_* | *df* | *F* | *p* | *η²_p_* | |
| MDD-ANX/PTSD (*n=46*) vs. HC-ANX/PTSD *(n=67)* |  |  |  |  |  |  |  |  |  |  |  |  |  |  |  |  | |
| Time | 4,424 | 59.65 | **<.001** | 0.360 | 5,520 | 24.88 | **<.001** | 0.193 | 5,515 | 18.62 | **<.001** | 0.153 | 5,510 | 33.43 | **<.001** | 0.247 | |
| Group | 1,106 | 43.72 | **<.001** | 0.292 | 1,104 | 0.17 | .68 | 0.002 | 1,103 | 5.05 | **.03** | 0.047 | 1,102 | 0.06 | .80 | 0.001 | |
| Sex | 1,106 | 1.84 | .18 | 0.017 | 1,104 | 0.94 | .33 | 0.009 | 1,103 | 0.29 | .59 | 0.003 | 1,102 | 1.99 | .16 | 0.019 | |
| Group x time | 4,424 | 2.89 | **.04** | 0.027 | 5,520 | 2.48 | .06 | 0.023 | 5,515 | 1.67 | .16 | 0.016 | 5,510 | 2.91 | .05 | 0.028 | |
| Sex x time | 4,424 | 1.51 | .21 | 0.014 | 5,520 | 2.21 | .09 | 0.021 | 5,515 | 3.39 | **.01** | 0.032 | 5,510 | 1.27 | .29 | 0.012 | |
| Time x group x sex | 4,424 | 1.76 | .16 | 0.016 | 5,520 | 0.41 | .74 | 0.004 | 5,515 | 0.27 | .88 | 0.003 | 5,510 | 0.44 | .67 | 0.004 | |
| MDD+ANX/PTSD *(n=54*) vs. HC-ANX/PTSD *(n=67)* |  |  |  |  |  |  |  |  |  |  |  |  |  |  |  |  | |
| Time | 4,452 | 64.81 | **<.001** | 0.364 | 5,560 | 23.69 | **<.001** | 0.175 | 5,555 | 18.52 | **<.001** | 0.143 | 5,555 | 39.16 | **<.001** | 0.261 | |
| Group | 1,113 | 57.67 | **<.001** | 0.338 | 1,112 | 0.00 | .99 | 0.000 | 1,111 | 2.73 | .10 | 0.024 | 1,111 | 0.19 | .66 | 0.002 | |
| Sex | 1,113 | 0.30 | .58 | 0.003 | 1,112 | 2.62 | .11 | 0.023 | 1,111 | 0.13 | .72 | 0.001 | 1,111 | 2.18 | .14 | 0.019 | |
| Group x time | 4,452 | 3.63 | **.01** | 0.031 | 5,560 | 2.35 | .07 | 0.021 | 5,555 | 3.54 | **.01** | 0.031 | 5,555 | 2.10 | .12 | 0.019 | |
| Sex x time | 4,452 | 1.66 | .17 | 0.014 | 5,560 | 1.43 | .24 | 0.013 | 5,555 | 4.01 | **.01** | 0.035 | 5,555 | 1.70 | .18 | 0.015 | |
| Time x group x sex | 4,452 | 0.80 | .50 | 0.007 | 5,560 | 0.69 | .56 | 0.006 | 5,555 | 0.95 | .42 | 0.009 | 5,555 | 1.16 | .32 | 0.010 | |
| *Note*: Repeated measures analyses of co-variance (rmANCOVA) with group (MDD vs. HC) and sex (female vs. male) as between-subject factors and time as within-subject factor. Autonomic nervous system responses controlled for age, pubertal status, BMI, smoking, sports, and psychological stress measure controlled for age and pubertal status. Because the assumption of sphericity was violated Greenhouse-Geisser corrections were applied in every analysis, but uncorrected degrees of freedom (df) are reported due to clarity. ANX/PTSD=anxiety and/or posttraumatic distress syndrome, RespHRV=Respiratory Heart Rate Variability, PEP=Pre-ejection period. | | | | | | | | | | | | | | | | |  |

| **Table S5**. Results of repeated measures analyses of covariance of the autonomic nervous system stress response in participants with major depressive disorder (MDD) compared to healthy controls (HCs) with psychological stress as covariate. | | | | | | | | | | | | | |
| --- | --- | --- | --- | --- | --- | --- | --- | --- | --- | --- | --- | --- | --- |
|  | Heart Rate | | | | RespHRV | | | | PEP | | | | |
|  | *df* | *F* | *p* | *η²_p_* | *df* | *F* | *p* | *η²_p_* | *df* | *F* | *p* | *η²_p_* | |
| Time | 5, 785 | 31.70 | **<.001** | 0.17 | 5, 780 | 21.93 | **<.001** | 0.12 | 5, 770 | 40.01 | **<.001** | 0.21 |  |
| Group | 1, 157 | <0.01 | .97 | 0.00 | 1, 156 | 6.11 | **.02** | 0.04 | 1, 154 | <0.01 | .99 | <0.01 |  |
| Sex | 1, 157 | 2.18 | .14 | 0.01 | 1, 156 | 0.06 | .81 | <0.01 | 1, 154 | 3.45 | .07 | 0.02 |  |
| Group x time | 5, 785 | 3.51 | **.02** | 0.02 | 5, 780 | 3.28 | **.01** | 0.02 | 5, 770 | 3.29 | **.03** | 0.02 |  |
| Sex x time | 5, 785 | 2.59 | .05 | 0.02 | 5, 780 | 3.59 | **<.01** | 0.02 | 5, 770 | 2.59 | .07 | 0.02 |  |
| Group x sex x time | 5, 785 | 0.81 | .81 | 0.01 | 5, 780 | 0.33 | .84 | <0.01 | 5, 770 | 0.84 | .45 | <0.01 |  |
| *Note.* Repeated measures analyses of covariance (rmANCOVA) with group (MDD vs. HCs) and sex (female vs. male) as between-subject factors, and time as within-subject factor. Analyses were controlled for age, pubertal status, body mass index, smoking, exercise and psychological stress. Where necessary, Greenhouse-Geisser corrections were applied, but uncorrected degrees of freedom (df) are reported here for clarity. PEP=pre-ejection period, RespHRV=respiratory heart rate variability. | | | | | | | | | | | | |  |

**
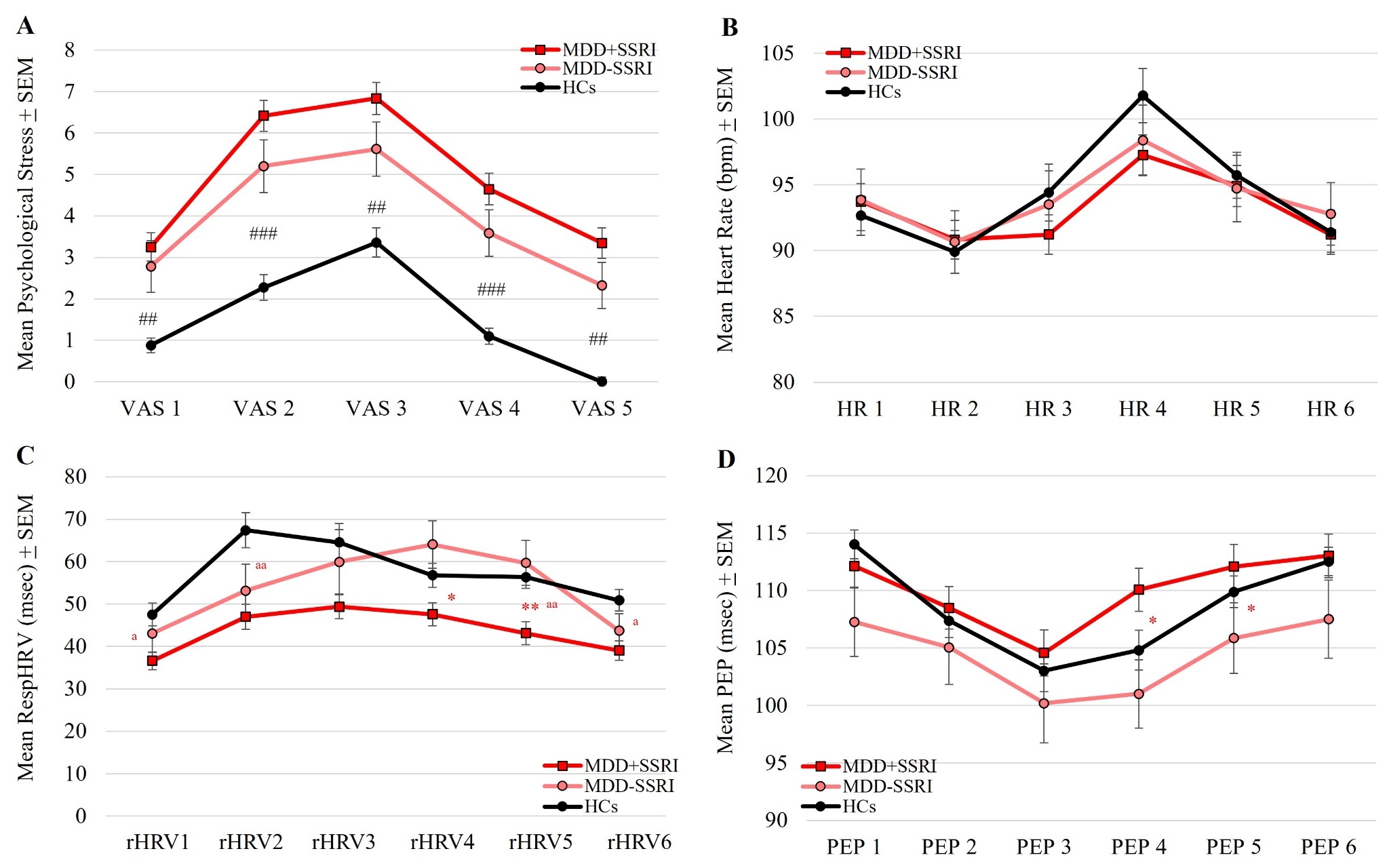
**

**FigS1.** Autonomic and psychological stress response during the Trier Social Stress Task in participants with major depressive disorder (MDD) with (+) and without (-) current selective serotonin reuptake inhibitors (SSRI) medication compared to healthy controls (HCs).

*Note.* Psychological Stress (A), HR (B), RSA(C), and PEP (D) responses to psychosocial stress. Significant group difference between MDD+SSRI and MDD-SSRI medication: **p*<0.05, ***p*<0.01. Significant group difference between both MDD groups and HCs: ##*p*<0.01, ###*p*<0.001. Significant difference between MDD+SSRI and HCs: ^a^*p*<0.05, ^aa^*p*<0.01. bpm=beats per minute, HR=heart rate, msec=milliseconds, PEP=pre-ejection period, rHVR=respiratory heart rate variability*.*

**References**

1. Kaufman J, Birmaher B, Brent D, et al (1997) Schedule for Affective Disorders and Schizophrenia for School-Age Children-Present and Lifetime Version (K-SADS-PL): initial reliability and validity data. J Am Acad Child Adolesc Psychiatry 36:980–988. https://doi.org/10.1097/00004583-199707000-00021

2. Harkness KL, Bruce AE, Lumley MN (2006) The role of childhood abuse and neglect in the sensitization to stressful life events in adolescent depression. J Abnorm Psychol 115:730–741. https://doi.org/10.1037/0021-843X.115.4.730

3. Wechsler D (2003) Wechsler Intelligence Scale for Children—Fourth Edition (WISC-IV). TX: Psychological Corporation, San Antonio

4. Wechsler D (2008) Wechsler Adult Intelligence Scale–Fourth Edition (WAIS–IV). TX: NCS Pearson 22, San Antonio

5. Organisation for Economic Co-operation and Development (1999) Classifying educational programmes: Manual for ISCED-97 implementation in OECD countries. Paris

6. Petersen AC, Crockett L, Richards M, Boxer A (1988) A Self-Report Measure of Pubertal Status: Reliability, Validity, and Initial Norms. J Youth Adolesc 17:117–133

7. Koopman-Verhoeff ME, Gredvig-Ardito C, Barker DH, et al (2020) Classifying Pubertal Development Using Child and Parent Report: Comparing the Pubertal Development Scales to Tanner Staging. Journal of Adolescent Health 66:597–602

8. Stiensmeier-Pelster J, Schürmann M, Duda K (2000) DIKJ Depressionsinventar für Kinder und Jugendliche

9. Kovacs M (1992) Children’s Depression Inventory Manual

10. Seddon JA, Rodriguez VJ, Provencher Y, et al (2020) Meta-analysis of the effectiveness of the Trier Social Stress Test in eliciting physiological stress responses in children and adolescents. Psychoneuroendocrinology 116:104582. https://doi.org/10.1016/j.psyneuen.2020.104582

11. Kirschbaum C, Pirke KM, Hellhammer DH (1993) The “Trier Social Stress Test”-A Tool for Investigating Psychobiological Stress Responses in a Laboratory Setting. Neuropsychobiology 28:76–81

12. Allen AP, Kennedy PJ, Dockray S, et al (2016) The Trier Social Stress Test: Principles and practice. Neurobiol Stress. https://doi.org/10.1016/j.ynstr.2016.11.001

13. Buske-Kkschbaum A, Wustmans A, Psych D, et al (1997) Attenuated Free Cortisol Response to Psychosocial Stress in Children with Atopic Dermatitis

14. Kajantie E, Phillips DIW (2006) The effects of sex and hormonal status on the physiological response to acute psychosocial stress. Psychoneuroendocrinology 31:151–178

15. Hellhammer J, Schubert M (2012) The physiological response to Trier Social Stress Test relates to subjective measures of stress during but not before or after the test. Psychoneuroendocrinology 37:119–124. https://doi.org/10.1016/j.psyneuen.2011.05.012

16. Dijk AE Van, Lien R Van, Eijsden M Van, et al (2013) Measuring Cardiac Autonomic Nervous System (ANS) Activity in Children. J Vis Exp 74:e50073. https://doi.org/10.3791/50073

17. De Geus EJC, Willemsen GHM, Klaver CHAM, van Doornen LJP (1995) Ambulatory measurement of respiratory sinus arrhythmia and respiration rate. Biol Psychol 41:205–227

18. Data Analysis and Management Software (DAMS) for the Vrije Universiteit Ambulatory Monitoring System (VU-AMS): Manual version v1.5. https://vu-ams.nl/support/

19. Riese H, Groot PFC, van den Berg M, et al (2003) Large-scale ensemble averaging of ambulatory impedance cardiograms. Behavior Research Methods, Instruments, & Computers 35:467–477

20. Sherwood A, Allen MT, Fahrenberg J, et al (1990) Methodological guidelines for impedance cardiography. Psychophysiology 27:1–23

21. Árbol JR, Perakakis P, Garrido A, et al (2017) Mathematical detection of aortic valve opening (B point) in impedance cardiography: A comparison of three popular algorithms. Psychophysiology 54:350–357. https://doi.org/10.1111/psyp.12799

22. Grossman P, Van Beek J, Wientjes C (1990) A comparison of three quantification methods for estimation of respiratory sinus arrhythmia. Psychophysiology 27:702–714

23. Van Lien R, Schutte NM, Meijer JH, de Geus EJC (2013) Estimated preejection period (PEP) based on the detection of the R-wave and dZ/dt-min peaks does not adequately reflect the actual PEP across a wide range of laboratory and ambulatory conditions. International Journal of Psychophysiology 87:60–69. https://doi.org/10.1016/j.ijpsycho.2012.11.001

24. Berntson GG, Lozano DL, Chen YJ, Cacioppo JT (2004) Where to Q in PEP. Psychophysiology 41:333–337. https://doi.org/10.1111/j.1469-8986.2004.00156.x
